# Molecular approach to the epidemiology of urinary schistosomiasis in France

**DOI:** 10.1371/journal.pntd.0009515

**Published:** 2021-07-06

**Authors:** Marie-Laure Gillardie, Oussama Babba, Caroline Mahinc, Maureen Duthel, Claire de Bengy, Clotilde Morineaud, Elisabeth Rivollier, Pierre Flori

**Affiliations:** 1 University of Saint-Etienne, GIMAP-EA-3064, Saint Etienne, France; 2 Parasitology and Mycology, department of Infectious Agents and Hygiene, University Hospital of Saint-Etienne, Saint-Etienne, France; 3 Department of Public Health, University Hospital of Poitiers, Poitiers, France; 4 Department PASS, University Hospital of Saint-Etienne, Saint Etienne, France; The University of Melbourne, AUSTRALIA

## Abstract

**Background:**

The diagnosis of urogenital schistosomiasis is based on the complementarity of serological technique and microscopic examination (ME). Between 2015 and 2019, the number of urinary schistosomiasis tests received in our laboratory increased sharply from 300 to 900 per year.

Therefore, we wanted to evaluate the reliability of urine microscopic examination (ME, reference and routine technique) from urine sample by comparing it to other techniques (antigenic technique and PCR). To this end, we optimized two real-time PCRs targeting respectively *Schistosoma haematobium (*Sh*)* and *Schistosoma mansoni (*Sm*)*.

**Methodology/Principal findings:**

914 urine samples from 846 patients suspected of urogenital schistosomiasis were prescribed and analyzed by PCR and also by antigenic technique for the first 143 samples. The antigenic technique evaluated was Schisto POC-CCA, Rapid Medical Diagnostics. These results (antigenic technique and PCR) were compared to ME which was performed from all urines.

The percentage of 14% (128/914) positive cases with the PCR technique and the percentage of 6.0% (54/914) positive cases with ME is significantly different (Chi 2 test, p<0.001). These 128 positive PCRs correspond to 120 different patients, 88.3% (106/120) of them were young migrants and 11.7% (14/120) were French patients returning from travel. Among these migrants, more than 75% (80/106) came from French-speaking West Africa.

In addition, the Schisto POC-CCA showed a specificity of 39% (46/117), too poor to be used as a screening tool in low or non-endemic areas.

**Conclusion/Significance:**

Targeted Sh and Sm PCRs in urine are reliable techniques compared to ME (reference technique). In view of our results, we decided to screen urinary schistosomiasis by direct ME always coupled by the PCR technique, which has shown better reliability criteria.

## Author summary

Urogenital schistosomiasis caused by *Schistosoma haematobium* (Sh) is a neglected tropical disease that is widespread in Africa and the Middle East. It is estimated that more than 100 million people are infected for a total population of 1600 million in these areas. Humans are infected during contact with freshwater by direct skin penetration by larvae. At present, the diagnosis of urogenital schistosomiasis is based on the complementarity of serological technique and urine microscopic examination (ME). ME remains the gold standard for the diagnosis of schistosomiasis in Africa. The aim of this work was to evaluate 2 new techniques and compare it to ME in order to improve the diagnosis from 914 urine samples. The first technique (Immunochromatographic antigen technique) showed a specificity of 39%, too poor to be used in low or non-endemic areas as in Europe. The second technique allowing (DNA based diagnostic -PCR) enabled us to increase the number of positive results by a factor of 2.4, from 54 diagnoses by ME to 128 by PCR. In view of our results, we decided to screen urogenital schistosomiasis by direct ME always coupled by PCR, which has shown better reliability criteria.

## Introduction

Schistosomiasis (or bilharzia) is one of the most important neglected tropical diseases (NTDs), affecting over 200 million people and causing 1.9 million disability-adjusted life-years (DALYs) [1].

*Schistosoma mansoni* (Sm) and *S*. *haematobium* (Sh), the predominating human species, cause intestinal schistosomiasis (mainly caused by Sm) and urogenital schistosomiasis (mainly caused by Sh).

Urogenital schistosomiasis affects over 100 million people in 53 countries in Africa and the Middle East [2,3]. This infection leads to chronic tissue inflammation with damage and complications when left untreated (as bladder carcinoma, obstructive uropathy and hydronephrosis…) [4]

In 2013, some cases of autochthonous transmission of urogenital bilharzia were reported in South Corsica in the Cavu Gorges [5]. Currently, in France, it is believed that the number of infected persons is increasing, mainly due to recent migratory flows making France and Europe a host country for many migrants [6].

The life cycle (see CDC life cycle for more information [7]) includes a freshwater mollusc as an intermediate host and human as the definitive host. Humans are infected through transcutaneous penetration of furcocercariae (specific larvae) released by the intermediate hosts during prolonged contact with freshwater. Subsequently, adult worms live in the capillary plexus of the bladder and genitourinary system. Eggs (Sh and Sm) are excreted in urine (Sh primarily) and in faeces (Sm primarily).

At present, screening for urogenital schistosomiasis is based on the complementarity of serological technique and direct microscopic examination (ME) of the urine centrifugation pellet [8]

ME remains the gold standard for the diagnosis of schistosomiasis, but it is a time-consuming and laborious technique that gives unreliable results. This lack of sensitivity may be due to circadian patterns and daily variations in egg excretion, and/or low parasite load, particularly in returned travelers [3].

Serological tests are very sensitive for schistosomiasis, but their specificity vary according to the technique and the population tested [8].

In non-endemic areas, serology is the most commonly used diagnostic approach because of good specificity in patients without prior exposure [8,9].

In endemic areas, serology-based screening is less suitable because of long-term exposure since childhood and it cannot differentiate an active infection of a past exposure, either medically treated or not [10,11]. Furthermore, the species involved and parasite load estimation is not possible with serological tests [12,13].

In recent years, there has been a great interest in the development of new molecular biology methods to improve the diagnosis of parasitic diseases [14,15]. The detection of *Schistosoma* DNA by PCR (Polymerase Chain Reaction) in urine has been described as a sensitive, specific and rapid detection tool, particularly important for low parasite load infections [16,17].

The objective of this work was to assess a PCR technique and compare it to different screening ImmunoChromatographic antigen Technique (ICT) and ME in order to improve our diagnostic approach to schistosomiasis, currently ME is used in the lab.

In this perspective, we have optimized two real-time PCRs for the diagnosis of schistosomiasis in urine samples: the first one specific for Sh and the second one specific for *S*. *mansoni* (Sm). The use of Sm PCR allows the detection of concomitant infections (Sm + Sh) which seem to be frequent several endemic African countries [18] and which are sometimes found only in the urine [19].

The Sh PCR targets *Dra1*, a 121 base-pair (bp) repeated sequence specific for Sh originally described by Hamburger *et al* [20]. Sm PCR targets *Sm1-7*, a 121 bp repeated sequence specific for Sm *described* by *Wichmann et al*. [21]. We tested these PCRs in urine samples to evaluate their species specificity and performance compared to microscopic screening. This study benefited from a very high urine recruitment, thanks to regional (Groupement hospitalier territorial “Loire and Nord Ardèche”) and national recruitment linked to a partnership with a national sized private laboratory.

## Material and method

### Ethics statement

The study has been approved by the Ethics Committee of the University Hospital of Saint-Etienne (No. IRBN 102 2019). The biological samples studied were received at the laboratory to carry out a schistosome search (presence of Schistosoma eggs in urine, on the 1st morning urination or on the 24-hour urine (French cost, 6.75 euros) on the basis of a medical prescription. All data were analyzed anonymously. According to the French law on public health [22], these types of protocols are exempt from the requirement of formal informed consent.

### Clinical samples

#### Negative samples

Fourty negative urine samples received at the laboratory between 01/05/2019 and 01/06/2019 were tested. These samples were morning urine from French patients who had never traveled to schistosomiasis endemic areas. All these samples were stored at +4°C before use.

#### Non-selected samples

**Fig 1:**
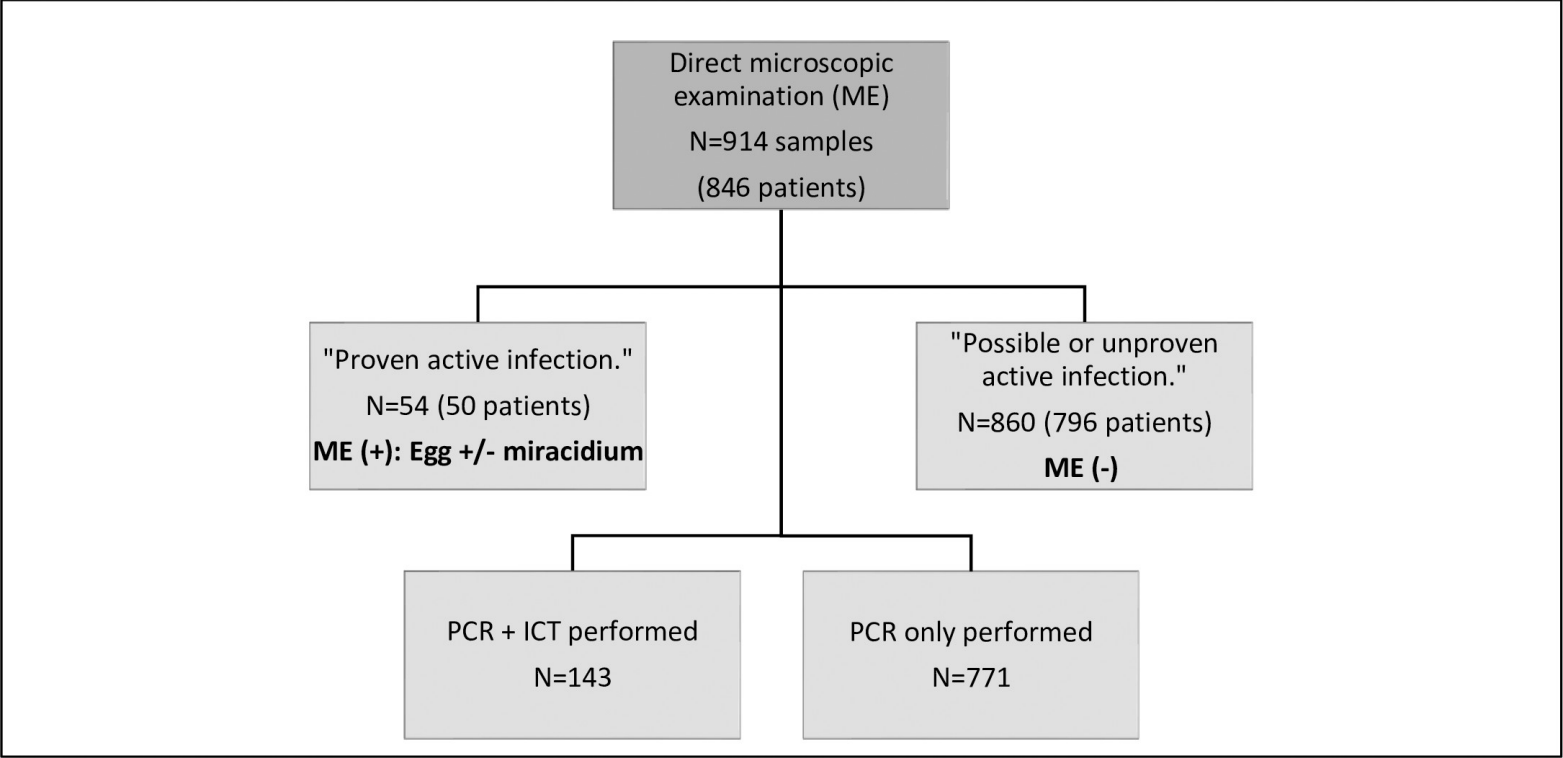
Comparison of techniques from 914 urine samples: study design and complementary techniques performed. ME (+): Positive Microscopic Examination, ME (-): Negative Microscopic Examination. ICT: Immuno-Chromatographic Test.

This corresponds to 914 urine samples (exhaustive sample) received at the laboratory from 01/05/2019 to 01/05/2020, and sampled for the detection of schistosome eggs (laboratory based diagnosis in France, cost 6.75 euros). All samples were stored at +4°C before use **(Fig 1).**

For the first 143 samples received, these are the techniques performed:

- A search for schistosome eggs by ME
- A specific antigen search by ICT (Schisto POC-CCA, Rapid test for qualitative detection of: Bilharzia (Schistosomiasis); Rapid Medical Diagnostics, Pretoria, South Africa)
- And a search for schistosome DNA using molecular biology techniques (PCR technique, see below).

For the remaining 771 urine samples

- A search for schistosome eggs by ME
- And a search for schistosome DNA using molecular biology techniques

### Classification of urine according to direct microscopic examination (ME)

On the basis of ME results, the urine samples were sorted into two groups: "proven active infection" and "possible active or unproven infection". Proven active infection" cases were defined as patients with eggs detected by urine ME with no past treatment. “Possible or unproven active infection" cases were defined by the absence of eggs detected by urine ME, associated, however, with a medical prescription justified by clinical and/or epidemiological arguments.

### Detection by direct microscopic examination (ME)

All the 914 samples were analyzed by ME. It was performed using an optical microscope by examining urine pellet samples (600 g/min for 6 minutes). The schistosomiasis diagnosis was confirmed by the presence of Sh eggs on the urine pellet after 4 slides read. We performed semi-quantification by dividing the results into 3 classes according to WHO criterion, (1) rare and few eggs (<50/10 ml), (2) numerous eggs (between 50 and 500 eggs/10 ml) and (3) very numerous eggs (> 500/10 ml) [23].

### ImmunoChromatographic antigen Technique (ICT) for the detection of schistosome-specific antigen (Schisto POC-CCA, rapid test for qualitative detection of Bilharzia (Schistosomiasis); Rapid medical diagnostics)

The schistosome-specific antigen test in urine was carried out in accordance with the manufacturer’s recommendations [24] on the first 143 urine samples received at the laboratory (May and June 2019). It should be noted that in case of a positive result, this technique does not allow the identification of the schistosome species in question.

### Detection by molecular biology technique (PCR technique)

The genomic DNA extraction was performed on the EasyMag NucliSENS (Biomerieux) automaton according to the manufacturer’s recommendations [25]. Extraction was performed on the urine pellet and supernatant from the same sample from "proven active infection" group and only on the centrifugation pellet from the "probable infection" group. From urine sample, 200μL of urine pellet and 500μL of supernatant was used for extraction. A 50μL eluate was recovered, and frozen if amplification was not performed immediately.

### Internal control

The extraction of each extract was controlled using beta-globin gene amplification (internal control), as described by Fabre *et al* [26]. The ß globin Forward and ß globin Reverse primers (ßGF 5’ TGA GTCTATGGGGACGCTTGA 3’; ßGR 5’ AAAAATTGCGGAGAAGAAAAAAA 3’) and the ß globin probe (ßGS 5’ TCCTGAGACTTCCACACTGAT GC 3’) labelled CY5—BHQ2, were used at final concentrations of 0.15 μmol/L for the primers and 0.15 μmol/L for the probe.

### Probes and selected primers

As specified by *Hamburger et al* [20], we chose to amplify the highly repetitive *Dra1* sequence of Sh (accession number DQ157698.1), and to use the following primers (Sh-FW 5’-GATCTCACCTATCAGACGAAAC-3 ’;

Sh-RV 5′-TCACAACGATACGATACGACCAAC-3 ′). The probe was chosen as described in 2013 by *Cnops et al* [17] (Sh-sonde 5′-TGTTGGTGGAAGTGCCTGTTTCGCAA-3 ’) and was marked 5 ’FAM, BHQ1 in 3’.

Based on the work of *Wichmann et al* [21], we have chosen to target the *Sm1-7de S* sequence.*mansoni* (GenBank accession number M61098), and to use the following primers (SRA1 5′-CCACGCTCTCTCGCAAATAATCT-3’; SRA2 5′-CAACCGTTCTATGAAAAATCGTTGT-3′), as well as the probe (SRP 5′-TCCGAAACCACTGGACGGTTTTGAT) marked 5’FAM, BHQ1 in 3’.

### Real-time amplification on lightcycler 480 version 1.2 (Roche)

During the same amplification, two distinct PCRs were performed, one specific for Sh, the other specific for Sm. The composition of the reaction mixtures of these two PCRs was identical.

The 25 μL reactions contained 5 μL of DNA, 12.5μL of Eurobio Probe Mix qPCR 2X Lo-Rox buffer, 0.25 μM of each primer, 0.3 μM of probe and 0.15 μM of internal control. The programmed cycle was as follows: 5 min at 95°C followed by 45 cycles of 10 seconds at 95°C and 30 seconds at 58° C. At each manipulation, a negative control and a positive control had to be amplified in parallel.

All specific exponential signal (3 successive cycles) was considered as specific.

### Egg detection signal and analytical variability of *S*. *haematobium* PCR

To determine analytical sensitivity of PCR technique, ME slides with a single identified schistosome egg were washed with sterile water. The washing water was then transferred to an Eppendorf. The previously washed slide was then re-examined through a microscope to objectify the egg recovery. The washing water containing the egg was extracted and amplified by PCR. This was done on 3 different samples.

### Statistical analysis

The specificity and sensitivity are calculated for the ICT only, because the concordance is poor with both the ME considered as the specific technique and the PCR considered as the sensitive technique. These results were obtained using the ME technique as reference.

We have compared different reliability criteria (% positive results) for the techniques used according to Chi-squared test (significance level of 0.05). We compared the average Ct according to t-Student test (significance level at 0.05). In order to evaluate the correlation between the semi-quantification on ME and the quantitative PCR Threshold Cycle (Ct) value, we determined the correlation coefficient and performed a Spearman test (significance level 0.05).

## Results

### The method validation of the PCR technique

#### Techniques specificities from our "negative" sampling

With the same PCR techniques, cross-reactivity has been previously studied by the work of *Guegan et al*. [19] using 34 positive samples with different protozoan or helminth parasites *(Toxoplasma*, *Plasmodium*, *Leishmania*, *Enterocytozoon bieneusi*, *Encephalitozoon Sp*, *Cryptosporidium sp*, *Endolimax nana*, *Blastocystis sp*, *Entamoeba hartmanni*, *Entamoeba dispar*, *Entamoeba histolytica*, *Entamoeba Coli*, *Dientamoeba fragilis*, *Giardia intestinalis*, *Enterobius vermicularis*, *Ascaris lumbricoides*, *Trichuris trichiura*, *Strongyloides stercoralis*, *Ancylostomidae*, *Hymenolepis nana*, and *Taenia sp*.). All of them (34/34) were found negative.

In addition, we tested 40 negative samples to ensure that, under our analytical conditions, there was no non-specific signal. There was a 100% analytical specificity (40/40 PCR negative).

#### Egg detection signal and analytical variability of *S*. *haematobium* PCR

**Fig 2:**
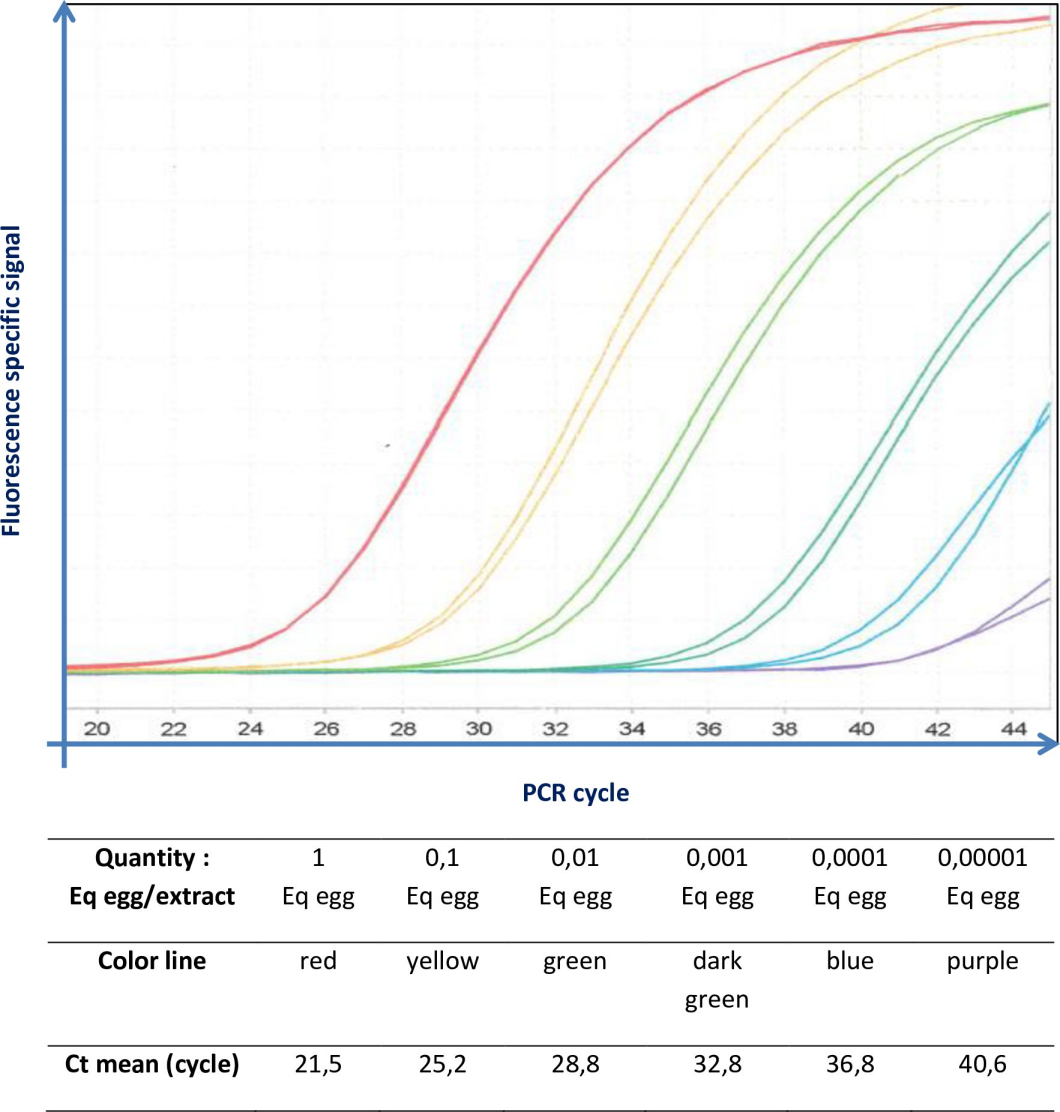
DNA Amplification signal curve of *S*. *haematobium* obtained from ranged dilutions between a quantity of one egg per extract and Equivalent (Eq) 0.00001 egg per extract. Characteristics of this PCR (standard curve): Efficiency = 1.83, slope = 3.80, Regression Coefficient (r^2^ = 0.99).

ME slides with a single identified schistosome egg were extracted and amplified as previously described. Thus, we determined that the presence of a single schistosome egg in sample corresponded to 20 +/- 2 Ct **(Fig 2).**

### Non-selected samples classification

A total of 914 urine samples from 846 patients were analyzed **(Fig 1).**

Out of these 914 samples, 54 samples were classified in the "proven active infection" group and 860 samples in the "possible or unproven active infection" group.

In a second step, all samples were analyzed by PCR. In addition, an ICT was performed on the first 143 urine samples.

### Performance of ICT compared to other techniques

In parallel with ME and PCR, an ICT was performed on the first 143 samples from both groups (proven active infection and possible or unproven active infection) **(Table 1).** Of the 143 ICTs performed, 19/21 (90%) were positive in the proven active infection group (ME+). However, from negative ME and negative PCR samples, 71/117 (bold number in Table 1) samples were positive and therefore not correlated with the result of ME. Considering ME as the reference technique, the evaluation of this test therefore shows a sensitivity of 90% and a specificity of 39%. In view of this lack of concordance between ICT, ME, and PCR, the evaluation of this test was stopped.

**Table 1:** Qualitative results of the ICT compared to PCR and direct microscopic examination (ME) results.

**Number Samples**			
	**ICT (+)**	**ICT (-)**	**TOTAL**
**ME (+) / PCR (+)**	19	2	21
**ME (-) / PCR (-)**	**71**	46	117
**ME (-) / PCR (+)**	3	2	5
**TOTAL**	93	50	143

### PCR performance and comparison with ME

#### Concordance between the PCR technique and ME

**Fig 3:**
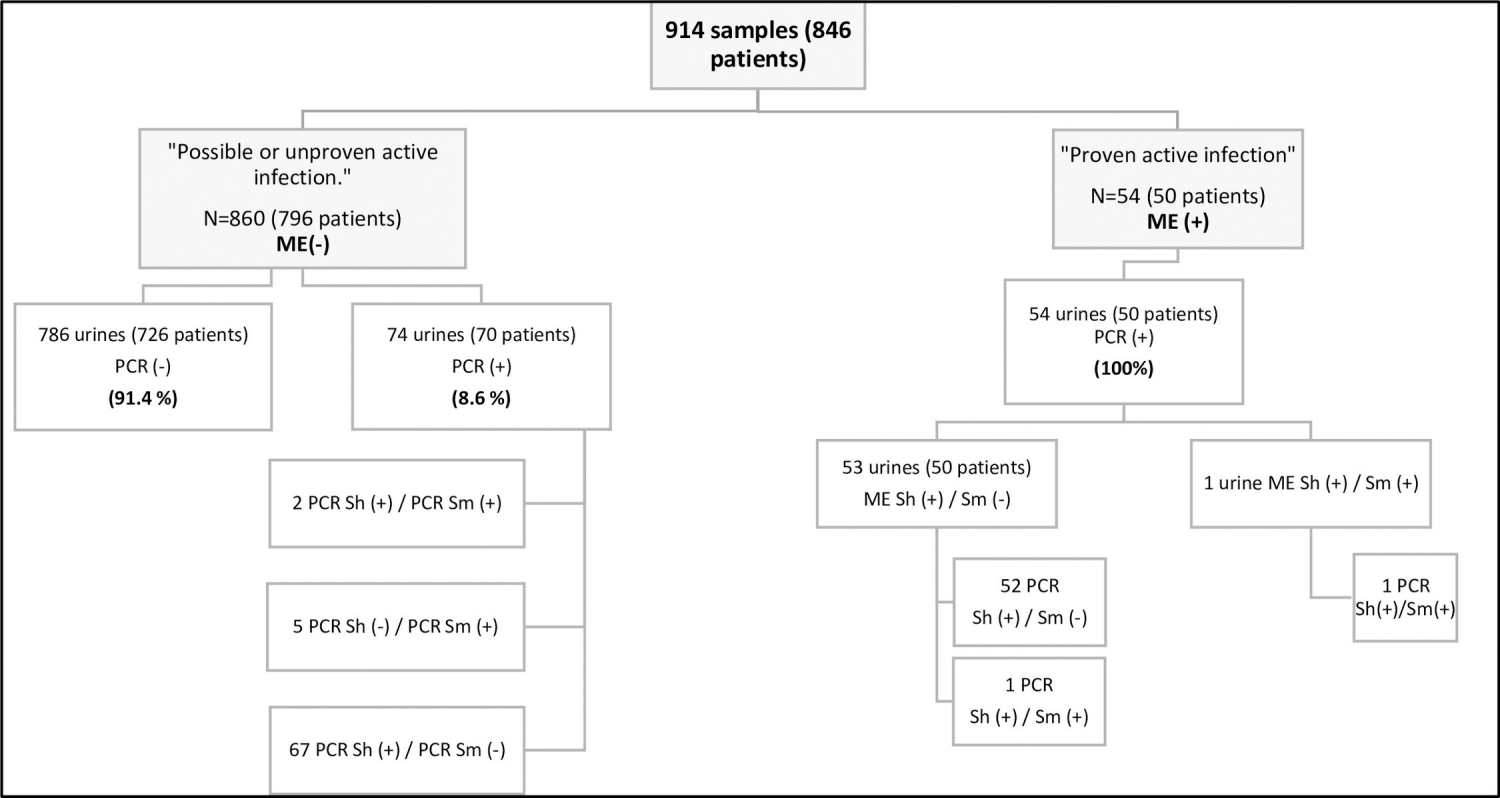
Comparison of direct microscopic examination (ME) and PCR results for the detection of *Schistosoma haematobium* (Sh) *and S*. *mansoni* (Sm) in urine specimens. ME (+): Positive Microscopic examination, ME (-): Negative Microscopic examination. PCR +: Positive PCR, PCR Sh +: Positive *Schistosoma haematobium* PCR, PCR Sm +: Positive *Schistosoma mansoni* PCR. PCR -: Negative PCR, PCR Sh -: Negative *Schistosoma haematobium* PCR, PCR Sm -: Negative *Schistosoma mansoni* PCR.

A comparison between ME and PCR technique was made on all samples. The results are shown in **Fig 3**. The percentage of 14% (128/914) positive PCR and the percentage of 6.0% (54/914) positive ME is significantly different (Chi squared test, p<0.001). Please note that all 54 positive ME samples also had a positive PCR signal. In total, for both groups, there is a 92% concordance between the results of ME and the PCR.

From 120 positive patients with PCR techniques (Sh and/or Sm), we found 111 positive patients (92.5%) with Sh PCR only, 5 positive patients (4.2%) with Sm PCR only and 4 patients (3.3%) with both positive PCR.

### Cycle of detection (Ct): Comparison and correlation between samples from proven active infections (Positive ME, semi-quantitation) and possible or unproven active infections (Negative ME)

The both PCRs Ct values (performed on urine pellet) were significantly higher in the 74 specimens in the "active infection possible or unproven" group than in the 54 "active infection proven" specimens. The average and standard deviations of the Ct values were 32.5 ± 5.8 for the "possible active or unproven active infection" group and 19.8 ± 4.2 for the "proven active infection" group (p<0.001, Student’s Test).

**Fig 4:**
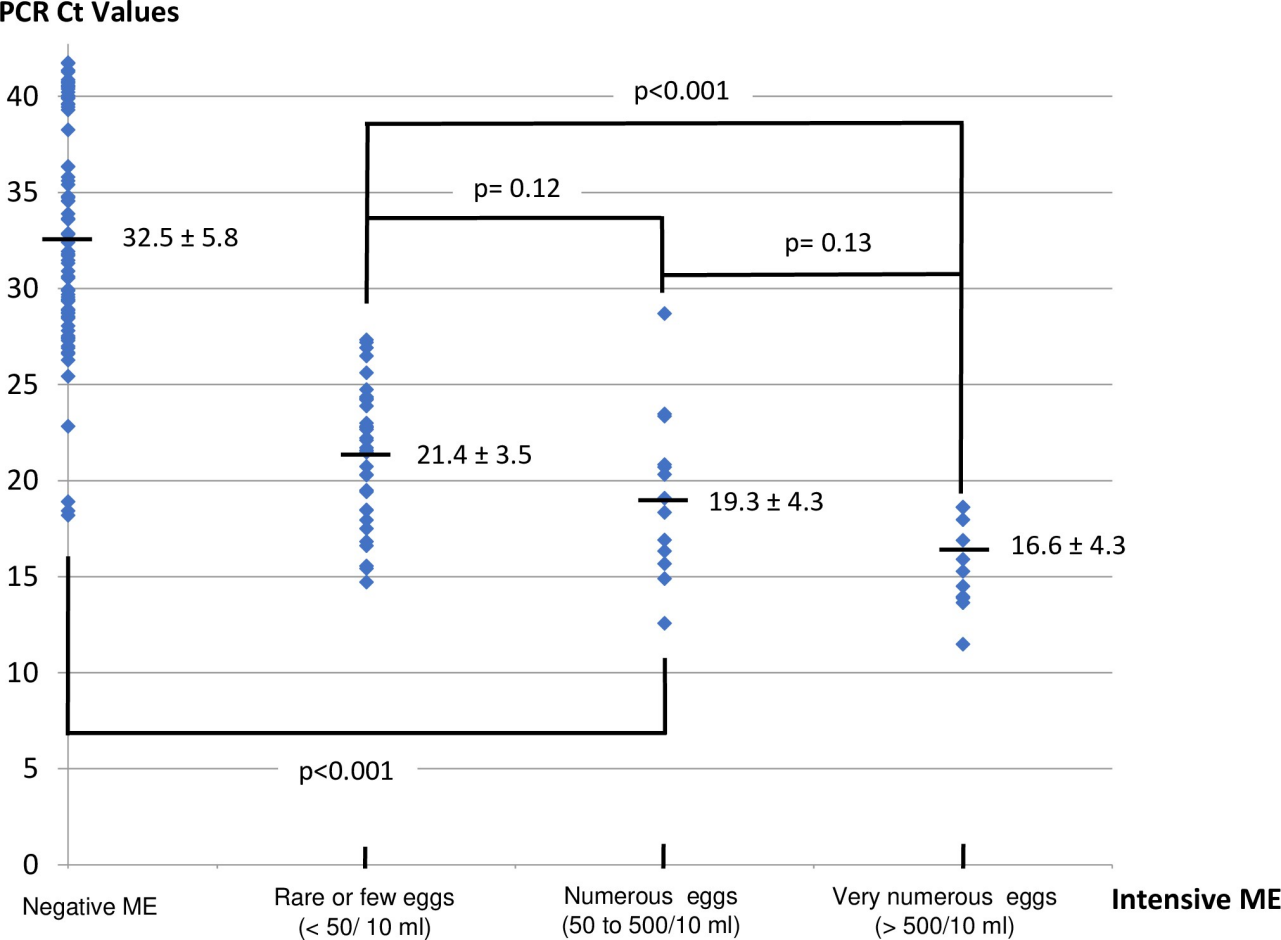
Comparison and correlation between samples from proven active infections (Positive ME, semi-quantitation) and possible or unproven active infections (Negative ME). r = 0.81, Significant correlation Spearman’s test, p<0.001.

In addition, we compared the PCR Ct values to the semi-quantitative results of the ME **(Fig 4).** The PCR Ct values are correlated with the results of ME (r = -0.81; p < 0.001, Spearman’s correlation test). The mean of both PCRs Ct values are proportional to the number of eggs detected by ME, 16.6 +/- 4.3 for urine with very numerous eggs, 19.3 +/- 4.3 for urine with numerous eggs, 21.5 +/- 3.5 for urine with rare and a few eggs and 32.5 +/- 5.8 for negative ME. This difference between positive and negative ME is significant (**Fig 4**, Chi squared test, p<0.001).

#### Comparison of pellet and supernatant detection cycles

**Fig 5:**
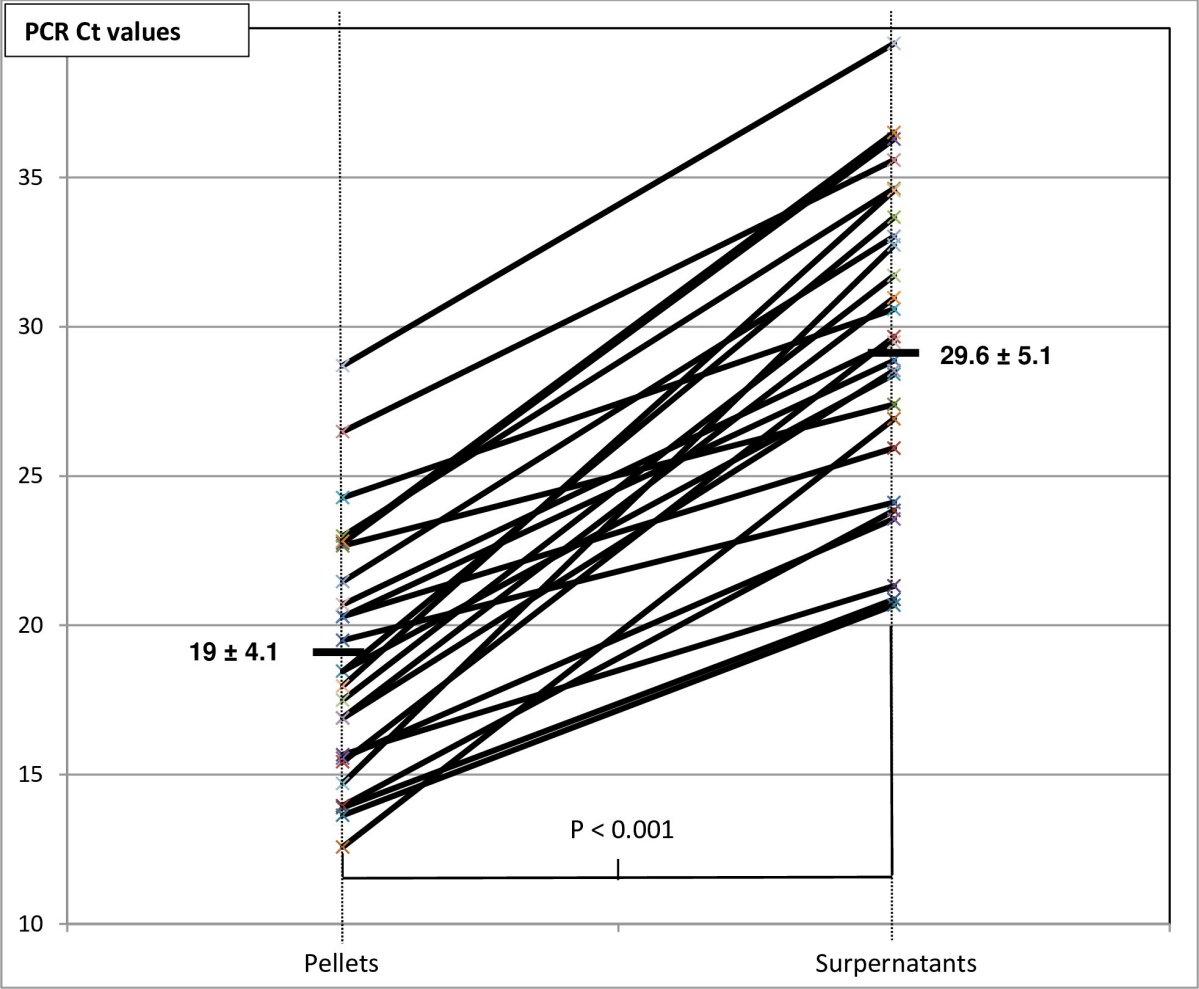
Comparison of Ct values between pellet and supernatant from the same urine sample (Student’s test for paired series, significant difference, p < 0.001). N = 26 samples with positive direct microscopic examination.

As of September 2019 until the end of our inclusion (May 2020), the samples sorted in the "proven active infection" group (n = 26), PCR was performed on both pellet and supernatant. In each sample, the supernatant Ct values were significantly higher than on the pellets (**Fig 5**). The average and standard deviations of Ct were 19 ± 4.1 for pellets and 29.6 ± 5.1 for supernatants. The difference between these paired samples is about 10 cycles on the average (t-Student test for paired series, significant difference, p<0.001).

### Epidemiological and clinical biological investigation based on positive PCR samples

An epidemiological investigation was conducted in all patients in the "proven active infection" group as well as in patients in the "possible active infection or unproven active infection" group in which Sh or Sm *was* detected by PCR.

Initially, we looked at the origin, age and sex of these patients. Among the 50 patients in the "proven active infection" group, 4 are of French origin (1 woman/3 men). The female was 6 years old, and the three male cases were 20, 30 and 49 years old. These 4 cases had travelled to endemic countries (the little girl had just returned from a stay in Senegal, and as for the men, 2 soldiers were back from Mali, one back from Madagascar). The other 46 patients were all African migrants (1 woman/45 men). The female case was 25 years old, and the average age of men was 20.6 (median 18 years of age).

Regarding the 70 patients in the "possible or unproven active infection" group in which the PCR showed specific DNA, 10 were of French origin (2 women/8 men); the two female cases were 43 and 52 years old, and the average age of men was 37.5 (median 36); all had travelled to endemic countries. The other 60 cases were all African migrants (2 females/58 males); the two female cases were 6 and 16 years old, and the average age of male cases was 19.9 (median 17).

**Fig 6:**
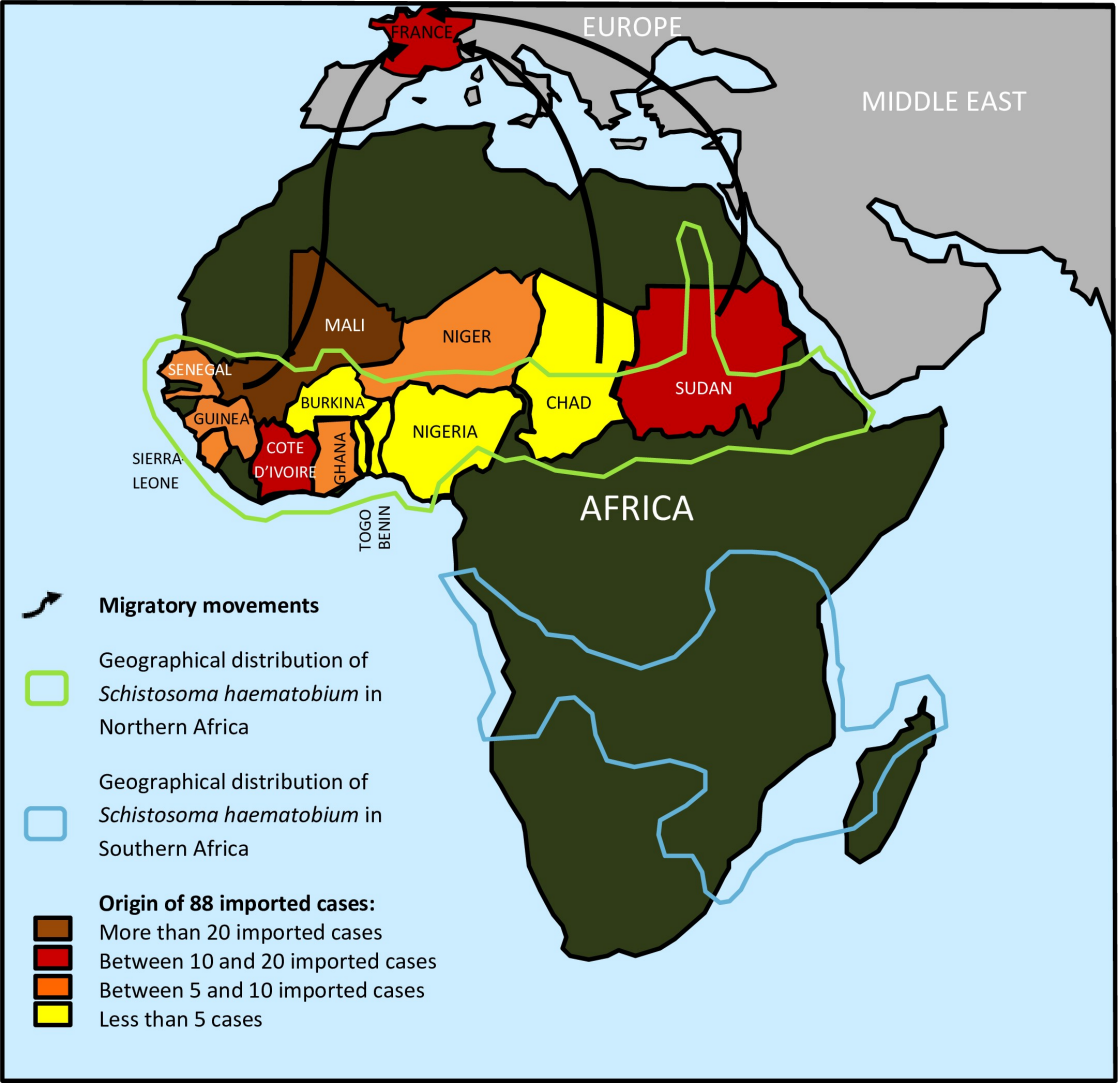
Global geographical distribution of urinary schistosomiasis cases (young African migrants living in France) diagnosed in our laboratory (Personally drawn geographic map with software Microsoft Word 2010).

The 106 African migrants with positive PCR (46 + 60) all came from countries in Northern Africa: 29 (27.4%) from Mali, 17 (16%) from Ivory Coast, 12 (11.3%) from Sudan, 9 (8.5%) from Senegal, 7 (6.6%) from Niger, 6 (5.7%) from Guinea, 5 (4.7%) from Sierra Leone and Ghana, 4 (3.8%) originated from Nigeria, Chad and Togo, 3 (2.8%) came from Burkina Faso and finally one case (0.9%) originated from Benin. The geographical origin distribution of these cases was represented the African continent map (**Fig 6**).

A clinico-biological analysis was carried out: the different clinical presentations that led to urinary schistosomiasis research **(Table 2)** shows that 70% of patients had macroscopic hematuria, 24% had urinary signs and 14% had a digestive problem. Only one patient (2%) had no clinical symptoms and no biologic secondary signs (normal urine cytobacteriological examination and normal complete blood count (CBC)).

**Table 2:** Clinical, biological signs and proposed treatment in patients with urinary schistosomiasis.

		**% (n = 50)**
**Macroscopic hematuria**		**70% (35)**
	Isolated macroscopic hematuria	66% (23)
	Macroscopic hematuria + urinary signs	20% (7)
	Macroscopic hematuria + digestive signs	14% (5)
**Urinary signs** (painful urination)		**24% (12)**
	Isolated urinary signs without hematuria	42% (5)
	Urinary signs + hematuria	58% (7)
**Digestive signs** (Digestive pain, constipation)		**14% (7)**
	Isolated digestive signs without hematuria	29% (2)
	Digestive signs + macroscopic hematuria	71% (5)
**Hypereosinophilia research** (CBC)		**74% (37)**
	Absence: eosinophil count < 0,5 G/L	49% (18)
	Moderate: eosinophil count (0,5 à 1,5 G/l)	27% (10)
	High: eosinophil count > 1,5 G/l	24% (9)
**Bilharzia serology**		**48% (24)**
	Positive serology (consistent with ME)	96% (23)
	Negative serology (inconsistent with ME)	4% (1)
**Microscopic hematuria**		**62% (31)**
	Microscopic hematuria	87% (27)
	Absence of microscopic hematuria	13% (4)
**Therapeutic management of infected patients**		
	Yes	84% (42)
	No (lost to follow-up our study)	16% (8)

*1 case had no clinical signs

The biological tests analysis associated with the ME urinary schistosomiasis search, shows that a test for hypereosinophilia (CBC) was requested in 74% of cases, bilharzia serology in 48% of cases, and a microscopic hematuria test by urine cytobacteriological examination was prescribed in 62% of cases.

When diagnoses were made, 84% of patients were treated medically, when 16% of them lost to follow up.

## Discussion

In this study, we explored the usefulness to assessing a PCR technique to detect the DNA of Sh and Sm and that of the ICT (Schisto POC-CCA) to improve the urogenital schistosomiasis diagnosis. We compared these results with those of ME, a technique which is considered as a reference by French government and WHO.

Concerning the evaluated antigenic technique (Rapid test for qualitative detection of: Bilharzia; Rapid Medical Diagnostics) and in view of our results and the bibliography [27–31], the diagnosis of Sh infection based solely on this technique does not seem to be reliable in low or non-endemic areas. Moreover, this test shows too poor specificity (39%). A preanalytic parameter can explain this data (lack of specificity): indeed we receive a lot of urines that have been stored from 1 to 4 days at +4°C related to our national recruitment. These preanalytical conditions are not optimized and may cause more frequently, false positives cases. In our condition, this test is therefore not recommended for the detection of Sh in urine and should be improved for this species.

As regards PCR techniques, these seem to be attractive tools which, due to their high sensitivity, would make it possible to meet this demand more precisely while being less restrictive in its implementation. PCR thus offers the advantage of reliable screening on one daily urination, rather than on a 24-hour urination, or on the first morning urination, as it is currently recommended in France. The PCR technique also has a benefit in terms of practicability because it allows a reliable response to a request for a urinary schistosomiasis research. Indeed in our study, a large number of samples do not meet all the criteria for analytical requirements (+4°C conserved samples, small volume). This is very beneficial for our laboratory, while the majority of requests come from non-specialized external laboratories with complex logistics that require the sample to pass through several sites before being analyzed and correspond to migrant patients for whom understanding and cooperation in care is sometimes difficult.

The two PCRs (Sh and Sm) chosen for our study are the most commonly used [19,20,32,33]. As shown by *Wichmann et al*. *the* high performance of these PCRs is linked to the presence of several copies of the targeted sequences, with a copy number in the order of 50 to 100 copies per genome [15,27], which allows very sensitive detection of the DNA of Sh and Sm.

In our study, all *Schistosoma spp* infections diagnosed by positive ME (54 urines), were confirmed by PCR and among the 860 negative ME, PCR detected 74 (8.6%) additional positive specimens. Compared to ME, PCR increased the number of positive results by a factor of 2.37 (Sh and Sm combined), increasing the number of positive results from 54 (6%) to 128 (14%).

Our study confirms the results of previous studies carried out in endemic areas showing a better sensitivity for these new tools [34,35]. The *Sm1-7* PCR carried out on 572 stool samples showed a positive examination number of 9.6% compared to 0.9% by ME [34], and the Sh *Dra1* PCR carried out on 401 urine samples showed a positive examination number of 36% compared to 25% by ME [35].

Moreover, to date, the "quantitative" signal of these two PCRs has never been studied. Moreover, this quantitative approach (not precisely evaluated for Sh and Sm) has been particularly interesting for the search for soil transmitted helminths, protozoa and *Schistosoma japonicum* showing highly variable levels of infection and co-infection depending on the parasite [36,37]. Therefore, we investigated whether PCR could provide quantitative information on parasite load. To this end, we determined that the presence of a single schistosome egg in the extracted/amplified sample corresponded to 20+/- 2 Ct. For positive ME samples, this “20 Ct threshold” was fully applicable. Fig 4 shows that the mean Ct values of the two PCRs are proportional to the number of eggs detected by ME: 16.6 ± 4.3 for urine with very numerous eggs detected by ME, compared with 21.4 ± 3.5 for urine with rare or a few eggs detected by ME.

For supernatants, a circulating DNA urinary load, corresponding to the presence of DNA strands and not a whole *Schistosoma* egg in the sample, was detected systematically. Indeed, several studies have shown that in urine, PCR detects mainly *Schistosoma* egg DNA, but it has been described that PCR signals may also correspond to transrenal nucleic acids of parasite degradation products detectable in urine as previously demonstrated for Sm [38,39] and other parasitic infections [40,41].

This theory proved to be consistent when we compared the Ct value between positive urine pellets (containing eggs) and supernatants (egg-free) on ME. All supernatants gave a positive signal with Ct >22, indicating the presence of Schistosoma DNA in these egg-free specimen parts.

Another great advantage of this PCR is that it allows precise identification of species, even when ME is negative or difficult (atypical or broken eggs, hybrids, etc.). This has a biological and clinical impact. Indeed, clinicians can adapt their management by setting up a targeted follow-up with the aim of identifying chronic complications induced by the *Schistosoma* species. In our study, the excellent identification of *Schistosoma* species by our two PCRs enabled us to identify a double infection with Sh and Sm in four African migrants (**Fig 3**).

The double positivity can be explained by two hypotheses. Firstly, we can suggest simultaneous infection of Sh and Sm, as is frequently observed in several endemic African countries and sometimes up to 50% of cases in some African regions [18]. The second hypothesis we can evoke is that of a hybrid egg (Sh and Sm) which would be detected by the positivity of the two PCR targets.

This second hypothesis is based on recent work that has shown the emergence of hybrid eggs in highly endemic areas. Based on these studies, different hybridizations have been identified, including hybridization between Sh and Sm [42–43] but also between human and bovine schistosomes [44–45].

In any case, early recognition of this infection remains essential because of possible chronic complications if left untreated. We can thus submit the hypothesis that all patients with a PCR amplification signal should be considered infected and therefore treated.

Previous screening work has shown a curiously low prevalence of schistosomiasis in a imported population in Europe, probably depending on the different screening tests used [46–49]. Indeed, among the 120 patients suffering from urinary schistosomiasis that we identified by PCR, only 42% (50 patients) were correctly detected by ME.

Of the patients we diagnosed with urinary schistosomiasis, only one was asymptomatic. All the others were symptomatic with macroscopic hematuria in 70% of cases, which could be isolated or associated with other less significant signs such as digestive pain and urinary burns.

The strength of our study is that we worked with a very large cohort of migrants, more than three-quarters of whom were young male refugees and asylum seekers (all from Africa). Eighty-six percent of our positive schistosomiasis cases were young men with an average age of 20 years (median 17 years), and were imported from the African continent, mainly from West francophone Africa, as reported in different studies [50–56].

Also, we are convinced that schistosomiasis PCR has brought two major developments to our screening practice. Firstly, it has made it possible to identify "non-excreting" schistosomiasis infections that could not be diagnosed by ME. Secondly, it allowed the diagnosis of "excretory" infections by recovering the false negative ME results, due to the low sensitivity and the small volume of urine received.

The PCR technique has many assets and drawbacks **(Table 3)** to be used in specialized laboratories in non-endemic areas. Indeed, the main drawback is related to the cost of this technique and this equipment and the main advantages are the possibility to perform a single sampling [19,57], the robustness of the result even if the volume of urine is low and/or preanalytical conditions are not optimized that this was shown in the current study, the reliability and the ability to detect co-infections (**Fig 3),** [19] and the possibility to detect low circulating urine DNA load (**Figs 4 and 5**) [58].

**Table 3:** Assets and drawbacks of the PCR technique.

Assets	Drawbacks
• Screening on a single sample • Ease of sample collection for the patient (only one micturition) • Facilitated screening for a population with poor compliance • Reliability of the result • Identification of co-infections and/or hybrids • Species identification • No random results related to the circadian cycle • Detection of urinary circulating DNA	• Expensive • Need expensive equipment and trained personnel • Refrigeration requirements of collected samples. • Low capacity to be made on demand, performed in series (once or twice a week and probably not daily)

## Conclusion

Urogenital schistosomiasis is an increasingly frequent but not very visible problem, as in France it mainly concerns African migrants (mainly West Africans) who have no or difficult access to healthcare systems.

Our work highlighted the high performance of targeted PCRs for Sh and Sm in urine from our recruitment. In fact, the PCR technique enabled us to increase the number of positive results by a factor of 2.4 (Sh and Sm combined), from 54 diagnoses of urinary schistosomiasis by ME to 128/914 by PCR.
